# Effect of infusion irrigation with different irrigating solutions on transient receptor potential vanilloid 5 and intra-articular inflammation in a post-traumatic osteoarthritis rabbit model

**DOI:** 10.1186/s40001-021-00491-0

**Published:** 2021-03-12

**Authors:** Xinghui Liu, Rong Chen, Liangbo Jiang, Xiangwei Li, Zhibo Sun

**Affiliations:** 1 Department of Traumatic Orthopedics, RenminHospital, Hubei University of Medicine, No.39 Chaoyang Road, Maojian District, Shiyan, 442000 Hubei China; 2 Department of Anatomy, Hubei University of Medicine, Shiyan, 442000 Hubei China

**Keywords:** Anterior cruciate ligament reconstruction, Post-traumatic osteoarthritis, Magnesium sulfate, Transient receptor potential vanilloid 5

## Abstract

**Background:**

The incidence of post-traumatic osteoarthritis (PTOA) after anterior cruciate ligament reconstruction (ACLR) is high, but there is still a lack of intra-operative preventive measures. This study aimed to evaluate the effect of different irrigating solutions continuous irrigation on intra-articular inflammation and cartilage degeneration.

**Methods:**

66 New Zealand rabbits were randomly divided into normal (N) group, no treatment (NT) group, sodium chloride (NaCl) group, magnesium sulfate (MgSO_4_) group, and calcium chloride (CaCl_2_) group. The right knee joint of the experimental group was utilized to construct the model of PTOA, and the left side was utilized as the normal control group. At different time points postoperatively, the blood concentration of hemoglobin and Mg2 + , the synovial fluid concentration of IL-1 β, TNF-α, tartrate-resistant acid phosphatase-5b (TRAP-5b), and Type II Collagen, the gene expression of IL-1 β and MMP-3, and the protein expression of TRPV5 and CaM were detected. Pearson′s linear correlation was employed to identify the possible relationship between the expression of TRAP-5b and the expression of IL-1β, IL-6, TNF-α, and Type II collagen. The hematoxylin and eosin staining (HE), Masson’s trichrome staining, and Alcian blue staining were performed at postoperative 35 days. Osteoarthritis Scoring (OA score) comprised categories including Alcian blue staining, cartilage histology, the cellular density of cartilage, degree of cell disintegration, and formation of chondrocyte cluster were blindly scored by trained researchers at postoperative 35 days.

**Results:**

There was no statistical difference (*P* > 0.05) in the hemoglobin concentration between different groups. The concentration of serum Mg^2+^ in the MgSO_4_ group was higher than that of the other three groups (*P* < 0.05) on the same day of operation, then gradually decreased. The expression of IL-1 β, IL-6, and TRAP-5b in synovial fluid increased 5 days after the operation, decreased at 15 days, and then increased again with time in the NT group, NaCl group, and NT group and NaCl group. At 35 days after the operation, the expression of IL-1 β, IL-6, TRAP-5b, and type II collagen in the MgSO_4_ group were lower than that in the other three groups (except group N) (*P* < 0.05).The correlation analysis results showed that the TRAP-5b levels correlated positively with IL-1 β, IL-6, TNF-α, and type II collagen concentrations. The histological examination revealed that the surface smoothness of cartilage, the morphology of chondrocytes, the arrangement of collagen fibers, and the density of proteoglycan in the MgSO_4_ group were better than those in other experimental groups. At 35 days postoperatively, the gene expression of IL-1 β and MMP-3 and the protein expression of CaM and TRPV5 in synovium in the MgSO_4_ group was lower than that in the NaCl group and CaCl_2_ group.

**Conclusion:**

Intra-operative irrigation with magnesium sulfate solution can inhibit the inflammatory factors and the expression of TRPV5, which can also reduce collagen loss and delay cartilage degeneration. Therefore, the use of magnesium sulfate in intra-operative irrigation may be an ideal choice to prevent PTOA.

## Background

Anterior cruciate ligament (ACL) reconstruction (ACLR) does not prevent the onset of post-traumatic osteoarthritis (PTOA) and its incidence is as high as 50% [1]. Despite restoring joint stability subjectively, ACLR surgery would not fully restore pre-injury joint biomechanics and microenvironment. Furthermore, dysregulation of the intra-articular inflammatory response following acute ACL injury may increase the risk of PTOA [2]. Unlike idiopathic osteoarthritis (OA), PTOA has a definite injury-time point, which means that interventions could theoretically be initiated at an early stage to prevent the progression of the disease [3]. Previous in vitro studies have found that exposure to normal saline (NS, 300 mOsm/L) will aggravate chondrocyte necrosis, and NS is often used as an irrigation fluid for ACLR surgery under arthroscopy. Compared to NS, hyperosmotic saline (HS, 600 mOsm/L) significantly reduced the chondrocyte death and the loss of type II collagen and aggrecan associated with scalpel-induced injury [4]. Besides, HS effectively mitigated the production of pro-inflammatory factors and degradative mediators [5]. However, HS used for arthroscopy was not associated with any detrimental effects on chondrocyte viability or tissue water content after 2 h of arthroscopic irrigation [6]. Nevertheless, HS reduced chondrocyte death during drilling compared to NS, whereas Mg^2+^ saline (5 mmol/L) reduced chondrocyte death compared to Ca^2+^ saline (5 mmol/L) [7].

Mg^2+^ is a natural antagonist of Ca^2+^ influx. The expressions of pro-inflammatory factors and degradative mediators in human cartilage and synovium explants were inhibited by Magnesium chloride (MgCl_2_, 20 mmol/L) in vitro [8]. At the same concentration (20 mmol/L), magnesium sulfate (MgSO_4_) could protect cartilage better than MgCl_2_ and reduce the loss of collagen II and proteoglycan, but the effect decreased with the increase of concentration (50 mmol/L and 100 mmol/L) [9]. The mechanism of MgSO_4_ inhibiting intra-articular inflammation is unclear, which may be related to the inhibition of Ca^2+^ influx. Transient receptor potential vanilloid 5 (TRPV5) is a subtype of TRPV, highly selective for Ca^2+^. TRPV5 can promote Ca^2+^ transport and be inhibited by Ca^2+^ endogenous antagonist Mg^2+^. The osmolality of the normal articular cavity is about 400 mOsm/L, however, the mRNA production of HIF-1 α and type II collagen in chondrocytes was highest when exposed to 380 mOsm/L medium [10].

In this study, one drilling hole in the femur of the rabbit knee joint was made to construct the model of PTOA [11], which was similar to the femoral side drilling in the ACLR. Then, the inflammatory factors and cartilage degeneration were observed by continuous irrigation with 400 mOsm/L magnesium sulfate solution (MgSO_4_), sodium chloride (NaCl), or calcium chloride solution (CaCl_2_) for 2 h. Herein, this study aimed to explore the effect of magnesium sulfate continuous irrigation on PTOA. The hypothesis was that intra-operative irrigation with magnesium sulfate solution can inhibit the inflammatory factors and the expression of TRPV5. We hope to provide new ideas for the prevention and treatment of PTOA at an early stage.

## Materials and methods

### Construction of PTOA model and liquid irrigation

All experimental protocols were approved by the Cornell Institutional Animal Care and Use Committee and followed the Animal Research: Reporting of In Vivo Experiments guideline. Female New Zealand rabbits aged 5–6 weeks (bodyweight = 2.4–3.0 kg) obtained from the Animal Experimental Center of Hubei University of medicine were used. All rabbits were kept in a temperature-controlled (22 ± 1 ℃) animal facility with a dark/light cycle of 12 h and free access to food and water.

**Fig.1:**
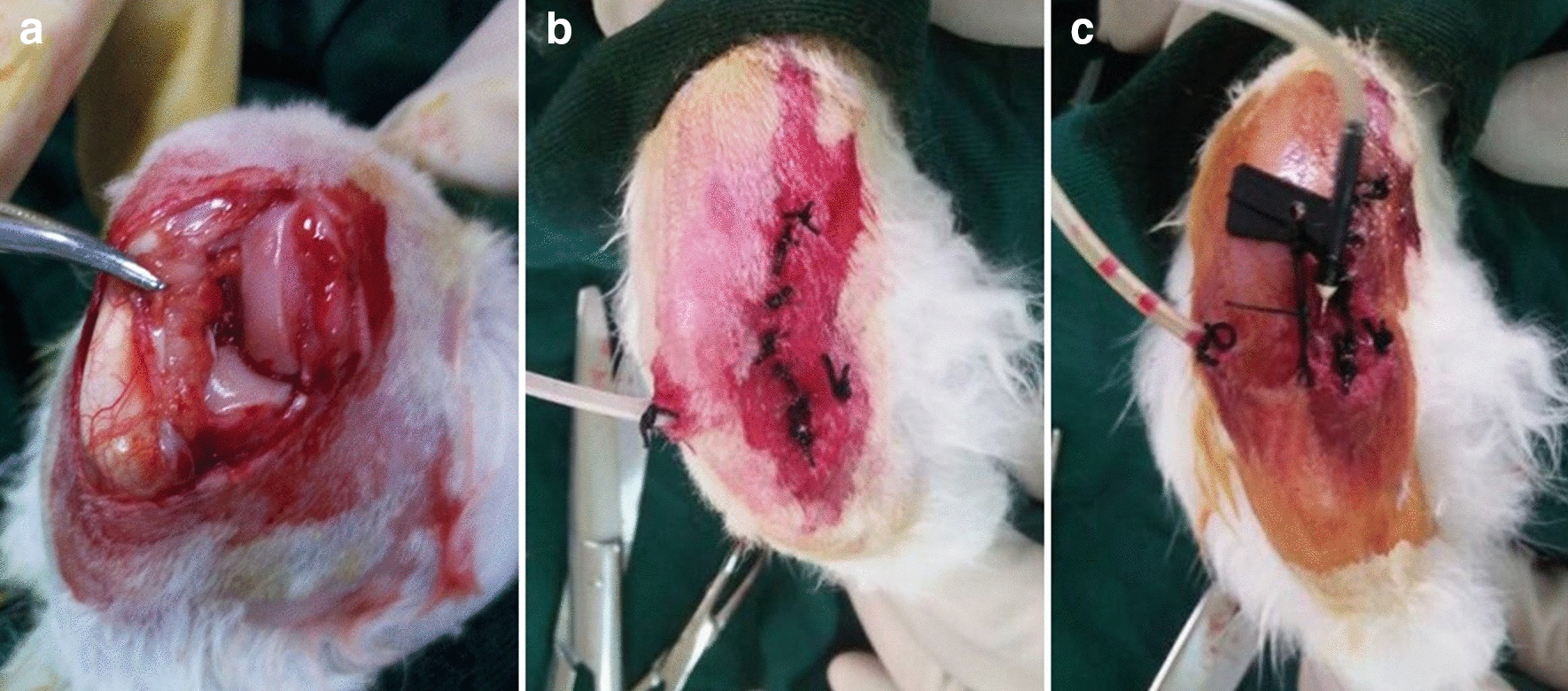
Construction of PTOA model and Infusion irrigation. **a** Exposure of femoral intercondylar fossa and drilling under the protection of sleeve. **b** Place the outlet pipe and fix it with sutures. **c** Insert the infusion needle as the water inlet pipe and fix it with sutures

Rabbit PTOA models were established regarding a method previously reported [11]. To induce PTOA, rabbits were first anesthetized with 3% pentobarbital through the auricular vein. An anterior medial curve incision (2–3 cm) was made on the right knee. Then a hole was drilled with a 3.2 mm drill bit under the protection of the sleeve. The direction was from the insertion point of the anterior cruciate ligament of the femoral intercondylar fossa obliquely to the lateral side and penetrated the contralateral cortex (Fig. 1a). The left knee of the same rabbit was taken as the normal control group. Then the inlet and outlet pipes were placed and fixed with sutures (Fig. 1b, c). Finally, different solutions were used for infusion irrigation under anesthesia for 2 h, the drip rate was 80 drops/min. Intramuscular injection of antibiotics (400,000 units of penicillin per kg of body weight) was performed 30 min before surgery and was continued for 3 days, and the skin was disinfected with iodophor for 7 days after surgery. All rabbits could eat and move in the cage freely after surgery. The general conditions of rabbits were recorded before and after the operation.

### Preparation of irrigation solutions

The osmotic pressure of all irrigation solutions was adjusted to 400 mOsm/L by adding sodium chloride and distilled water. Sodium chloride solution (NaCl): sodium chloride only, magnesium sulfate solution (MgSO_4_): containing 20 mmol/L magnesium sulfate, calcium chloride solution (CaCl_2_): containing 20 mmol/L calcium chloride.

### Experimental design

**Fig.2:**
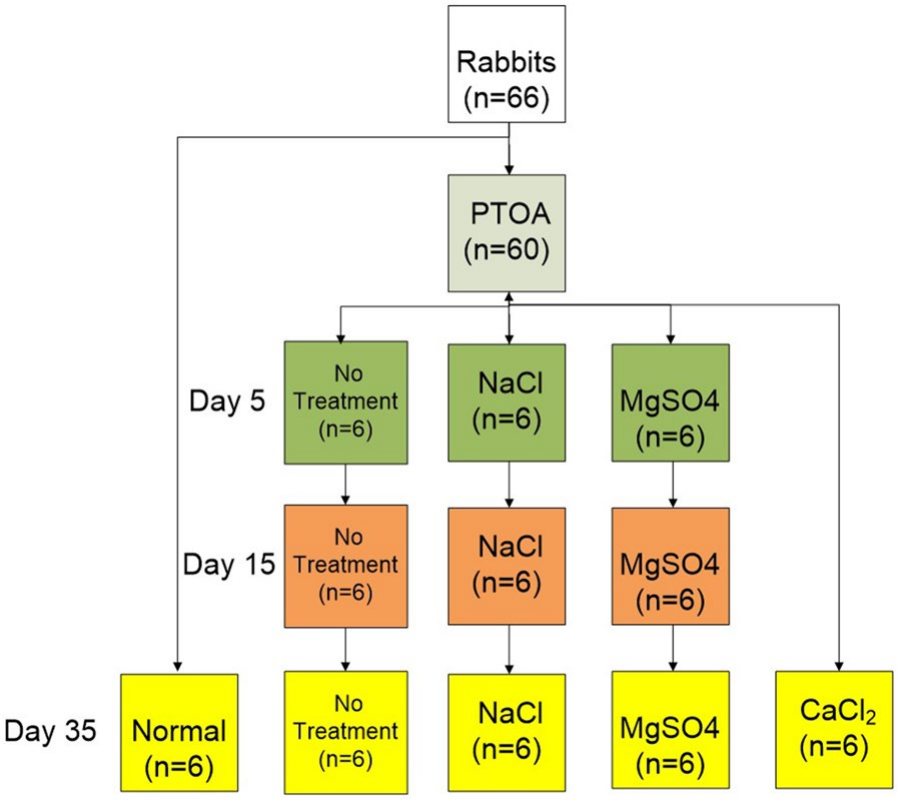
Experimental design. Rabbits were randomly allocated to six groups: normal group (*n* = 6), no treatment group (*n* = 18), NaCl group (*n* = 18), MgSO4 group (*n* = 18), and CaCl2 group (*n* = 6). Six rabbits were fed normally for 35 days as normal group. The other 60 rabbits were used to construct the PTOA model on their right knee joints

Rabbits were randomly allocated to five groups: normal group (*n* = 6), no treatment group (*n* = 18), NaCl group (*n* = 18), MgSO4 group (*n* = 18), and CaCl2 group (*n* = 6) (Fig. 2). Normal group (N): six rabbits were fed normally for 35 days. The right knee joint of the rest rabbits was used to construct the model of PTOA, and the left knee joint was used as the normal control. In no treatment group (NT), NaCl group, and MgSO4 group: on the 5th, 15th, and 35th day after operation and irrigation, six rabbits were killed, respectively. The data of the NT group on the 5th and 15th day were used as the positive control group. CaCl_2_ group: CaCl2 was used as a TRPV5 promoter, so the time point was just set to 35 days only.

### Blood hemoglobin and Mg^2+^ detection

At 0, 15, and 35 days after the operation, 1 ml blood was collected from rabbit ear vein, and hemoglobin and Mg^2+^ concentration were detected by colorimetry according to the steps of the detection kit (SinoBestBio, Shanghai, China).

### IL-1 β, TNF-α, TRAP-5b, and type II collagen detection by ELISA

At 5, 15, and 35 days after the operation, 1 ml physiological saline was first injected into the rabbit’s knee joint to collect the synovial fluid. The joint was then flexed and extended 10 times before the fluid was collected [12]. The collected fluid was immediately centrifuged at 3000 rpm for 15 min. The sera were then aliquoted and stored at – 80 ℃ until use. The concentration of IL-1 β, TNF-α, tartrate-resistant acid phosphatase-5b (TRAP-5b), and Type II collagen in the synovial fluid was determined by enzyme-linked immunosorbent assay (ELISA) according to the manufacturer’s protocol (SinoBestBio, Shanghai, China).

### Histology and osteoarthritis scoring (OA score)

At 35 days after the operation, the medial condyle of the femur was fixed at 4% buffered formaldehyde, dehydrated, paraffin-embedded, and sectioned into 4-μm-thick slides. Hematoxylin and eosin staining (HE) was done to evaluate the surface of the cartilage and cell morphology. Masson’s trichrome staining was performed to assess the accumulation and alignment of collagen. Alcian blue staining was used to examine the distribution of proteoglycan. All staining followed the manufacturers’ instructions. Stained slides were examined under an optical microscope (Olympus, Japan) and captured using a digital CCD camera. The modified histological OA scores were blindly scored by a medical staff member according to previously described scoring systems [13], which would provide a comprehensive evaluation of the cartilage tissue and chondrocyte status. The OA score comprised categories including Alcian blue staining (score: 0–6, according to staining in the hyaline cartilage), cartilage histology (score: 0–6, according to erosion in the hyaline cartilage), the cellular density of cartilage (score: 0–3, according to decrease in cells), degree of cell disintegration (score: 0–3, according to loss of columns), and formation of chondrocyte cluster (score: 0–3, according to number of clusters) as published [14].

### IL-1 β, MMP-3, and TRPV5 detection by quantitative RT-PCR

Synovium tissues were harvested at 35 days postoperatively. Total RNA was extracted from these tissues and mRNA levels determined by qRT-PCR. For this purpose, equal amounts of RNA were subsequently transcribed into the first-strand cDNA, then amplified in the presence of specific primers (Table1) and SYBR Green/ROX qPCR Master Mix. GAPDH was included as an internal loading control. Reactions were conducted in a CFX96 qRT-PCR detection system (Bio-Rad). The specific amplification of the interest gene was validated by the melting curve. The relative gene expression was derived from the CT values of the amplification curve using the 2^−ΔΔCт^ method.

**Table 1:** Primers used for qRT-PCR determinations

Gene	species	GenBank ID	Strand	Sequence 5′–3′
IL-1β	Rabbit	NM_001082201.1	Forward	GCC GAT GGT CCC AAT TAC AT
			Reverse	ACA AGA CCT GCC GGA AGC T
MMP-3	Rabbit	NM_001082280.1	Forward	GCC AAG AGA TGC TGT TGA TG
			Reverse	AGG TCT GTG AAG GCG TTG TA
GAPDH	Rabbit	NM_001082253.1	Forward	GGAGGCAGGGATGATGTTCT
			Reverse	TGTTTGTGATGGGCGTGAA

### TRPV5 and CaM detection by western blot

Synovium tissues were harvested at 35 days postoperatively. Total proteins were extracted from these tissues with lysis buffer. Protein concentrations were calculated using the BCA protein concentration determination method. Approximately 30 µg of protein was resolved on a sodium dodecyl sulfate-polyacrylamide gel and transferred to a polyvinylidene difluoride membrane. Membranes were incubated with specific antibodies against TRPV5, CaM(1:1500, AmyJet Scientific, Wuhan, China), and GAPDH(1:2000, KangChen, China) overnight at 4 °C. Then secondary antibody treated with an Immobilon Western Chemiluminescent HRP substrate was added for 1 h. The expression of the protein was detected using the ECL chemiluminescence method. Protein expression was calculated with ImageJ software.

### Statistical analysis

All data were analyzed using SPSS version 22.0 software. Measurement data were presented as mean ± standard derivation, and the comparisons were examined by repeated-measures ANOVA, two-way ANOVA, and one-way ANOVA, the homogeneity of variance was examined by the LSD method, and the missing variance was examined by the Dunnett-t method. Pearson's linear correlation was employed to identify the possible relationship between the expression of TRAP-5b and the expression of IL-1β, IL-6, TNF-α, type II collagen. A p-value of less than 0.05 was considered statistically significant.

## Results

### General conditions of rabbits

There was no significant change in body weight (not shown) before and after the operation (*P* = 0.872). The swelling and redness of the incision disappeared in 1 week after the operation, and there was no exudation. The wound healed well and no infection occurred 2 weeks after the operation. There was no vomiting, convulsion, and other symptoms in all groups during and after the operation.

### Changes of hemoglobin and Mg^2+^ concentration in blood at different time points after operation

**Fig.3:**
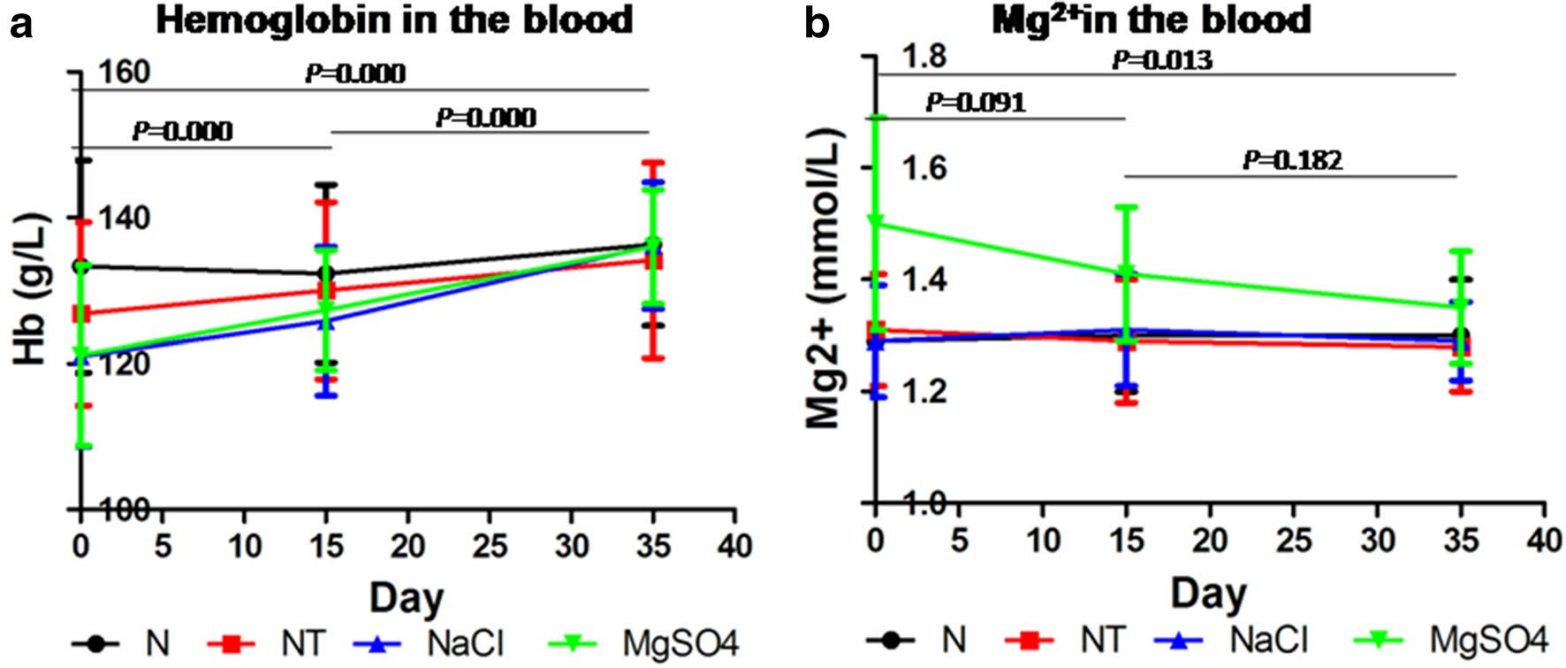
Changes of hemoglobin and Mg^2+^ concentration. **a** The hemoglobin concentration in the MgSO_4_ group and the NaCl group increased gradually with time. **b** The concentration of Mg^2+^ in the MgSO_4_ group decreased with time

Although the hemoglobin concentration in the MgSO_4_ group and the NaCl group was lower than that in the N group on the same day of operation, there was no statistical difference (*P* = 0.760), and it returned to normal level at 35 days after the operation (Fig. 3a). The concentration of serum Mg^2+^ in the MgSO_4_ group was higher than that of the other three groups (*P* = 0.047) at the same day of operation, then gradually decreased. There was no significant difference in Mg^2+^ concentration among the other three groups (*P* = 0.126) (Fig. 3b).

### Expression of IL-1 β, IL-6, TNF-α, TRAP-5b, and type II collagen at different time points after operation

**Fig.4:**
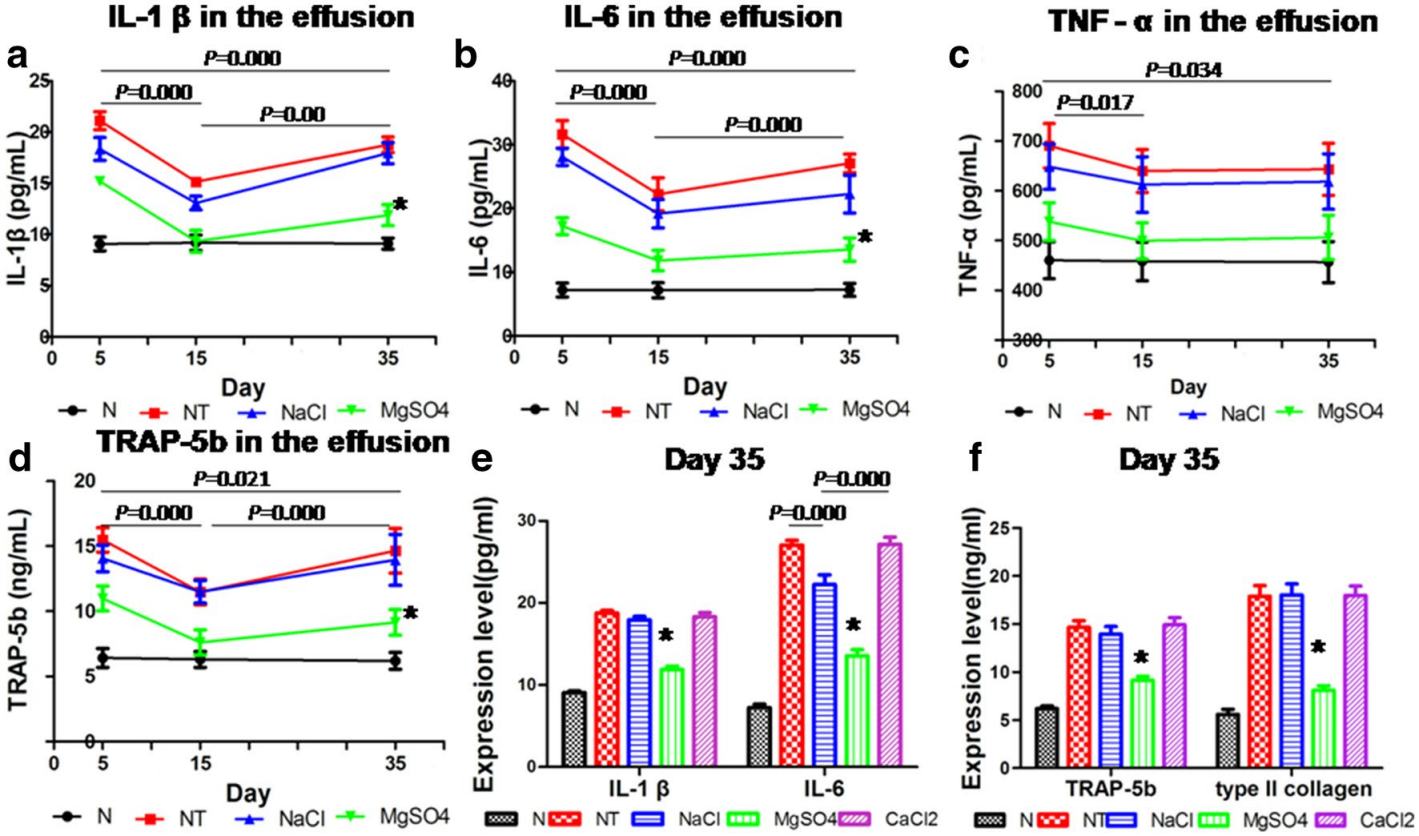
The expression of IL-1 β, IL-6, TNF-α, TRAP-5b and type II collagen in the synovial fluid. **a**, **b**, **c** and **d**, at 5, 15, and 35 days after operation, the expression of IL-1 β, IL-6, TNF-α and TRAP-5b in group N, NT, NaCl and MgSO_4_. **e** and **f**, At 35 days after operation, the expression of IL-1 β, IL-6, TRAP-5b and type II collagen in group N, NT, NaCl, MgSO_4_ and CaCl_2_ *P < 0.05

There were significant differences (*P* = 0.000) between different groups at each time point in the expression IL-1 β, IL-6, and TRAP-5b, and there were also significant differences (*P* = 0.000) between different times in each group, but the expression of TNF-α was not significantly different between different times or groups (*P* = 0.889). The expression of IL-1 β, IL-6, and TRAP-5 increased at 5 days after the operation, decreased at 15 days, and then increased again with time in the NT group, NaCl group, and NT group and NaCl group (Fig. 4a, b, d). At 35 days after operation, the expression of IL-1 β, IL-6, TRAP-5b, and type II collagen in the MgSO_4_ group were lower (*P* < 0.05) than that in the other three groups (except group N) (Fig. 4e, f). The expression of IL-1 β, IL-6, TRAP-5b, and type II collagen in the CaCl_2_ group was the highest. There was no significant difference compared with the CaCl_2_ group, NT group, and NaCl group, except that the expression of IL-6 in the CaCl_2_ group was significantly different from that in the NaCl group (*P* = 0.000). At 35 days after the operation, the expression of IL-1 β, TRAP-5b, and type II collagen was not significantly different between the NT group and NaCl group (*P* > 0.05), however, the expression of IL-6 in the NaCl group was lower than that in the NT group (*P* = 0.000), and the expression of IL-6 in the CaCl_2_ group was significantly higher than that in NaCl group (*P* = 0.000) (Fig. 4e, f).

### The correlation between the expression of TRAP-5b and the expression of IL-1 β, IL-6, TNF-α and type II collagen

We then conducted a correlation analysis between the expression of TRAP-5b and the expression of IL-1 β, IL-6, TNF-α, and type II collagen. The results showed that the TRAP-5b levels correlated positively with IL-1 β, IL-6, TNF-α, and type II collagen concentrations (Table 2).

**Table 2:** Correlation analysis between the expression of TRAP-5b and the expression of IL-1 β, IL-6, TNF-α and type II collagen

Inflammatory factors	r	P value	95% CI
IL-1 β	0.913	0.000	0.223–0.674
IL-6	0.905	0.000	0.048–0.302
TNF-α	0.774	0.000	–0.007–0.006
Type II collagen	0.849	0.000	0.414–0.676

### Histological observation of cartilage sections in each group at 35 days postoperatively

**Fig.5:**
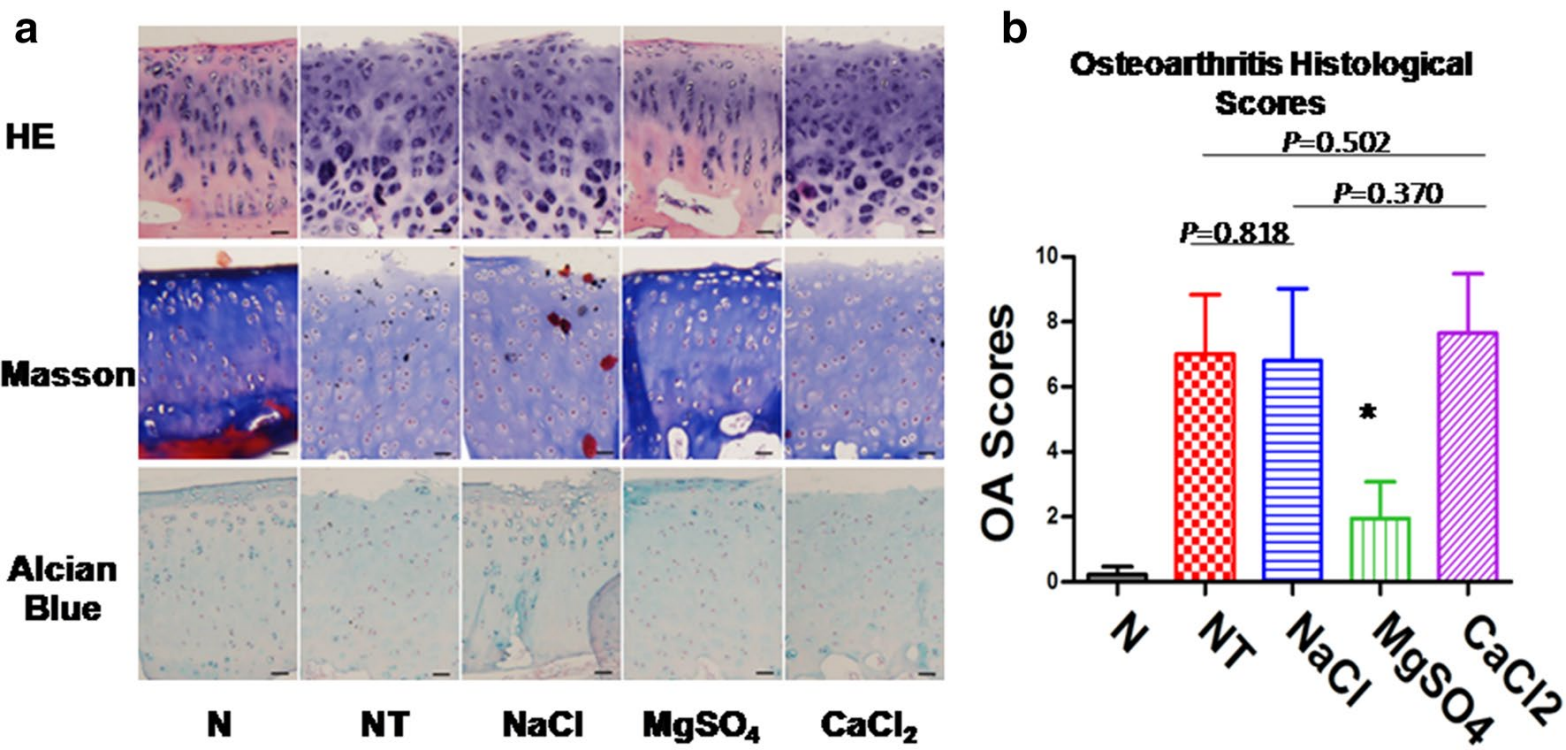
Histological observation of cartilage sections and OA score. **a** From top to bottom, the typical results of HE staining, Masson’s trichrome staining, and Alcian blue staining. Compared with the other three groups (except group N), the surface of cartilage tissue in the MgSO4 group was smoother and maintained good chondrocytes, with a good arrangement of collagen fibers and higher density of proteoglycan. **b** The OA score of each group. The OA score of the MgSO4 group was substantially lower than that of the remaining three groups (except group N), and the OA score of the CaCl_2_ Group was the highest, but there was no statistical significance when compared with group NT or NaCl (*P* > 0.05). Black scale bar = 50 μm. *P < 0.05

HE staining results showed that the surface of cartilage tissue in the MgSO4 group was smoother and the chondrocytes maintained well. However, there were different degrees of irregularity and disorder of chondrocytes in group NT, NaCl, and CaCl_2_. The MgSO_4_ group had a good arrangement of collagen fibers in Masson’s trichrome staining, and the density of proteoglycan was higher in Alcian blue staining, while the other three groups had different degrees of collagen fiber arrangement disorder and proteoglycan loss (Fig. 5a). The OA score of the MgSO_4_ group was substantially lower than that of the remaining three groups(Except group N) (*P* < 0.05), and the OA score of the CaCl_2_ Group was the highest, but there was no statistical significance when compared with group NT (*P* = 0.502) or NaCl (*P* = 0.370) (Fig. 5b).

### Gene and protein expression in synovial tissue in each group at 35 days postoperatively

**Fig.6:**
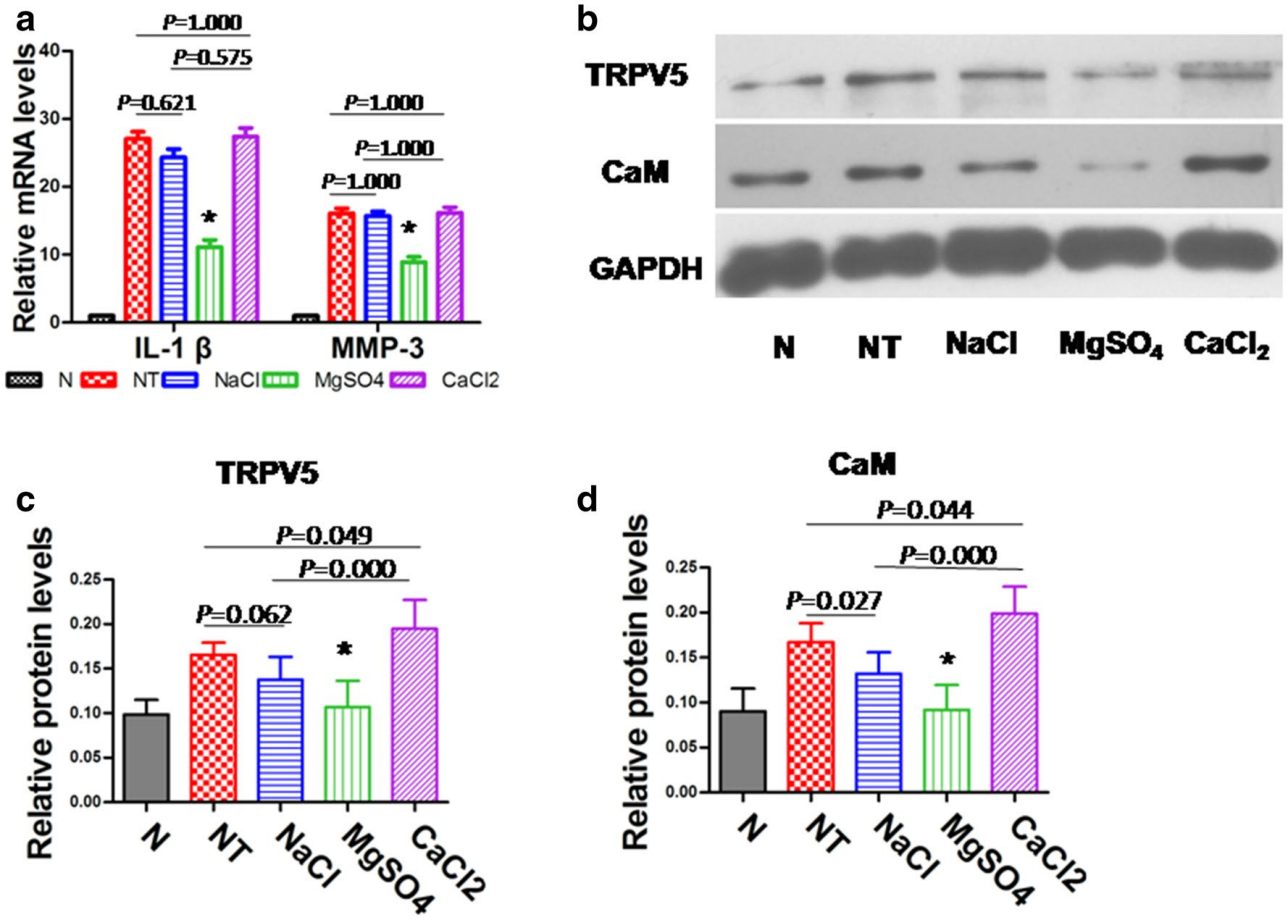
The gene and protein expression in synovial tissue in each group at 35 days after operation. **a** Relative gene expression of IL-1 β and MMP-3. **B **CaM and TRPV5 protein banding. **c**, **d** Relative protein expression of the CaMand TRPV5.*P < 0.05

At 35 days after operation, the expression of IL-1 β and MMP-3 genes in synovium of the MgSO_4_ group was lower than that in the other three groups (except group N) (*P* < 0.05), while the expression of those in the CaCl_2_ group was the highest, but there was no significant difference compared with NT group and NaCl group (*P* = 0.575, *P* = 1.000) (Fig. 6a). The expression of TRPV5 and CaM protein in synovium of the MgSO_4_ group was lower than that of the other three groups (except group N) (*P* < 0.05). However, the expression of TRPV5 and CaM protein in the CaCl_2_ group was the highest, which was significantly different from that in the NT group and NaCl group (*P* = 0.049, *P* = 0.000, *P* = 0.044, *P* = 0.000) (Fig. 6b, c, d).

## Discussion

Although ACLR can restore joint stability, it cannot reduce the occurrence of PTOA, the intra-articular inflammation may play a key role in the evolution of the disease [2, 15, 16]. Early chondrocyte necrosis leads to the exposure of cell scaffold and the weakening of stress tolerance. The intra-articular inflammation accelerates the necrosis of chondrocytes and aggravates the degradation of the cartilage matrix, leading to cartilage degeneration [17]. Therefore, to prevent the occurrence of PTOA, we should pay attention to avoid the early necrosis of chondrocytes during operation. At present, NS is often used for continuous irrigation in ACLR, however, exposure to NS will aggravate chondrocyte necrosis [4, 7]. Based on the fact that MgSO_4_ not only has an anti-inflammatory effect, but also has a protective effect on cartilage [8, 9, 18], our research group proposed to use MgSO_4_ solution instead of NS as the continuous irrigation fluid. Firstly, we used the femoral intercondylar drilling to construct the model of PTOA, which was similar to the femoral ligament tunnel of ACLR. It did not damage the stability of the joint and involve the load-bearing articular surface [11]. Therefore, it is a good model for the study of intra-articular inflammation. It can eliminate the mechanical factors such as joint instability and joint surface unevenness to aggravate intra-articular inflammation. Secondly, we used the infusion lavage method to simulate continuous irrigation under arthroscopic surgery. Finally, we used an osmotic pressure of 400 mOsm/L (normal intra-articular osmotic pressure) irrigation solution for continuous irrigation for 2 h under good anesthesia conditions. In this study, continuous irrigation for 2 h after drilling without tourniquet resulted in hemoglobin loss, but no anemia. In addition, the level of magnesium ion in blood of the MgSO_4_ group increased immediately after operation, but there was no significant difference compared with other groups. Thus, it can be seen 400 mOsm/L magnesium sulfate (20 mmol / L) continuous irrigation for 2 h is safe and reliable. Besides, we found that compared with the NaCl group, the MgSO_4_ group can reduce the intra-articular inflammatory reaction, reduce the secretion of IL-1 β, IL-6, and TNF-α in the joint cavity effusion. The results are consistent with those reported by other scholars [8, 19]. Surgery or other trauma can lead to increased intra-articular inflammation, which can be reflected in our results. However, whether it is caused by trauma or subsequent invents, under the same treatment conditions, magnesium sulfate group could inhibit intra-articular inflammation better compared with other groups.

Both intra-articular IL-6 and IL-1 β can induce the production of metalloproteinases (MMPs), which degrade the extracellular matrix of cartilage, and induce osteoclast (OC) differentiation, eventually cause articular cartilage damage [20]. TRAP is one of the cell markers of OC and its precursor cells. OC secretes cathepsin K to degrade articular cartilage, which plays an important role in cartilage destruction and degeneration [21, 22]. In this study, we found that TRAP secretion was higher in the NT group than in the MgSO_4_ group at different time points. However, compared with the NT group, the expression of TRAP in the NaCl group was lower at 5 days, and the expression continued to increase with time, then there was no significant difference between the two groups at 35 days. Continuous irrigation can reduce OC in the articular cavity, but OC can still exude from bone marrow through the drilling hole in the femur. Therefore, TRAP increased gradually in the NaCl group. Moreover, previous studies have shown that the serum Mg^2+^ concentration of osteoporosis women is lower than that of normal women, and believe that the lack of Mg^2+^ can lead to OC activation [23]. Our results suggest that MgSO_4_ can inhibit intra-articular inflammation and inhibit OC activation. Inflammatory factors such as IL-1 β, TNF-α and IL-6 can activate OC through receptor activator of nuclear factor-κ B ligand (RANKL) [24]. Besides, we also found that TRAP was positively correlated with IL-1 β, IL-6, TNF-α, and type II collagen in joint effusion.

Previous in vivo and in vitro studies have found that MgSO_4_ can not only inhibit intra-articular inflammation but also reduce the loss of type 2 collagen and proteoglycan, which could protect cartilage [8, 9, 18, 19]. In our research, histological sections results showed that the smoothness, chondrocyte morphology, collagen fiber arrangement, and proteoglycan density in the MgSO_4_ group was better than that in other groups. TRPV5 is a subtype of TRPV, highly selective for Ca^2+^. TRPV5 can promote Ca^2+^ transport and be inhibited by Ca^2+^ endogenous antagonist Mg^2+^ [25]. Also, TRPV5 can induce extracellular Ca^2 +^ influx and promote the phosphorylation of calmodulin-dependent protein kinase II (CaMK II), thus increasing the intra-articular inflammatory response and promoting chondrocyte apoptosis [26]. TRPV5 possesses a tightly regulated negative feedback mechanism, where the ubiquitous Ca^2 +^ binding protein calmodulin (CaM) directly binds to the intracellular TRPV5 C-terminus. When the level of Ca^2 +^ increases, they form a tight complex and promote the influx of Ca^2 +^ [27]. We found that the expression of TRPV5 and CaM increased in the CaCl_2_ group, but decreased in the MgSO_4_ group. Besides, the expression of IL-1 β and MMP3 in the synovial tissue in the CaCl_2_ group was increased, while that in the MgSO_4_ group was decreased. The increase of extracellular Ca^2 +^ concentration triggers the activation of NLRP3 inflammatory bodies in monocytes through the calcium-sensitive receptor (CaSR) and promotes the release of IL-1 β, which aggravates the intra-articular inflammation [28]. However, MgSO_4_ is a natural antagonist of Ca^2 +^ influx, can inhibit the expression of TRPV5 [25, 29], and can block endothelial IL-1 β secretion [30].

This study still has some shortcomings, such as a small number of samples, less observation time. We should extend the observation time and increase the relevant indicators for the detection of cartilage growth in the future.

## Conclusion

Continuous irrigation with irrigating solution containing magnesium sulfate (20 mmol / L) for 2 h during the operation is safe. Intra-operative irrigation with magnesium sulfate solution can inhibit the inflammatory factors and the expression of TRAP, and its possible mechanism is to inhibit the expression of TRPV5. Besides, it can reduce collagen loss and delay cartilage degeneration, thus preventing PTOA. Therefore, the use of magnesium sulfate in intra-operative irrigation may be an ideal choice to prevent PTOA.

## Data Availability

The datasets generated and analyzed during the current study are available from the corresponding author on reasonable request.
